# Safety and Tolerability of Accelerated Low-Frequency Repetitive Transcranial Magnetic Stimulation Over the Primary Motor Cortex–A Pilot Study

**DOI:** 10.3389/fnins.2022.793742

**Published:** 2022-03-18

**Authors:** Melina Engelhardt, Jana Kimmel, Giovanni Raffa, Alfredo Conti, Thomas Picht

**Affiliations:** ^1^Department of Neurosurgery, Charité – Universitätsmedizin Berlin, Berlin, Germany; ^2^Einstein Center for Neurosciences, Charité – Universitätsmedizin Berlin, Berlin, Germany; ^3^Division of Neurosurgery, Dipartimento di Scienze Biomediche, Odontoiatriche e delle Immagini Morfologiche e Funzionali (BIOMORF), University of Messina, Messina, Italy; ^4^Dipartimento di Scienze Biomediche e Neuromotorie (DIBINEM), Alma Mater Studiorum University of Bologna, Bologna, Italy; ^5^IRCCS Istituto delle Scienze Neurologiche di Bologna, Bologna, Italy; ^6^Cluster of Excellence, Matters of Activity, Image Space Material, Humboldt-Universität zu Berlin, Berlin, Germany

**Keywords:** transcranial magnetic stimulation, neuromodulation, accelerated, low-frequency, motor cortex

## Abstract

Low-frequency repetitive transcranial magnetic stimulation (rTMS) is capable of inducing changes in the functional organization of underlying brain regions, however, often at the cost of long stimulation protocols over several weeks. As these protocols can be difficult to implement in clinical settings, the aim of the present pilot study was to show the feasibility and safety of an accelerated low-frequency rTMS protocol applying multiple sessions daily. To this purpose, nine healthy subjects received 14 sessions of rTMS (1 Hz, 30 min, 110% RMT) to the hand motor hotspot. Subjects received stimulation for either 14 days once daily [classical rTMS (c-rTMS)], 7 days twice daily (accelerated rTMS; a-rTMS), or sham stimulation for 14 days once daily (s-rTMS). Daily stimulation sessions in the a-rTMS group were delivered with a 90-min break in between. In total, 74% of rTMS sessions in the c-rTMS group, 89% in the a-rTMS group, and 98% in the s-rTMS group were free of any side effects. Brief headaches and fatigue in stimulated muscle groups were the most frequent side effects. All side effects were reported to be at maximum mild and of short duration. Thus, accelerated low-frequency rTMS of the motor cortex seems to be a safe and feasible method, previously shown to induce a functional reorganization of the motor system. By shortening treatment duration in days, this approach can potentially make rTMS protocols more accessible to a wider range of patients.

## Introduction

Low-frequency repetitive transcranial magnetic stimulation (rTMS) is capable of inducing changes in functional organization of underlying brain regions, however, often at the cost of long stimulation protocols over several weeks [see Lefaucheur et al. (2020) for an overview of common protocols]. Thus, although theoretically offering promising treatment approaches, these lengthy protocols are difficult to implement in clinical settings where patients are only seen for limited times. Accelerated rTMS protocols applying multiple rTMS sessions daily have been shown equally effective compared to classical protocols for high-frequency stimulation (Modirrousta et al., 2018). However, studies investigating the potential and safety of accelerated low-frequency rTMS in the motor domain are still scarce.

Chae et al. (2004) investigated the safety of four daily low-frequency rTMS sessions [1 Hz, 110% resting motor threshold (RMT)] for 5 days in patients with Tourette’s syndrome. Each session lasted for 10 min with a 50-min break between sessions. Flamez et al. (2016) applied rTMS twice daily (1 Hz, 90% RMT) for 5 days in patients with Parkinson’s disease. During each session, both hemispheres were stimulated for 16 min consecutively followed by a break of at least 1 h. Only one study (Chae et al., 2004) reported the occurrence of mild, temporary headaches in a small percentage of cases, but no severe adverse effects were observed in either study. Since the publication of these studies, rTMS paradigms have been further intensified and single session stimulation durations were increased—a change that is also visible in the recently updated safety guidelines (Rossi et al., 2021). These intensified protocols again warrant the need for a detailed analysis of safety and tolerability of the intervention. The aim of the present pilot study was to provide preliminary evidence for the feasibility and safety of such an accelerated low-frequency rTMS protocol for the motor domain.

## Materials and Methods

### Participants

Nine healthy subjects (age: mean = 25.4 years, range = 22–31 years; 1 female) provided their written informed consent for this study. Subjects were recruited *via* advertisements in local student groups. All subjects were naïve to rTMS and completed a screening form for contraindications to TMS and MRI before inclusion. TMS exclusion criteria were history of epilepsy (also within the family), migraine, tinnitus, history of neurological or psychiatric illness, pregnancy, and intake of prescription drugs within the past 14 days. Further, subjects were excluded from receiving an MRI scan if they had permanent makeup, tattoos, or metallic implants including any form of intrauterine devices. The study was designed as single-blinded sham-controlled randomized trial, approved by the local ethics committee and conducted in accordance with the Declaration of Helsinki.

### Neuronavigated Transcranial Magnetic Stimulation

A T1-weighted structural MRI (TR = 2,500 ms; TE = 2.22 ms; TI = 1,000 ms; flip angle = 8°; voxel size = 0.8 × 0.8 × 0.8 mm; 208 slices) was used as subject-specific navigational dataset for the TMS. Neuronavigated TMS was applied using a Nexstim NBS 5 stimulator (Nexstim, Helsinki, Finland) with a figure-of-eight coil (outer diameter of 70 mm). Motor evoked potentials were recorded from the first dorsal interosseous muscle of the non-dominant hand *via* disposable Ag/AgCl surface electrodes (Neuoline 700; Ambu, Ballerup, Denmark) attached in a belly-tendon fashion. The ground electrode was attached to the left palmar wrist. Muscle activity of the target muscle was monitored to remain below a maximum tolerated baseline activity of 10 μV. Further, muscle activity was monitored throughout the session to identify any sign of epileptic activity. The hotspot was recorded for each subject as the point, electric field direction, and angulation, consistently eliciting the largest motor evoked potentials. For this point, the RMT was determined before the first rTMS session using the systems inbuilt automated threshold hunting algorithm (Kumpala et al., 2009).

### Repetitive Transcranial Magnetic Stimulation

**FIGURE 1:**
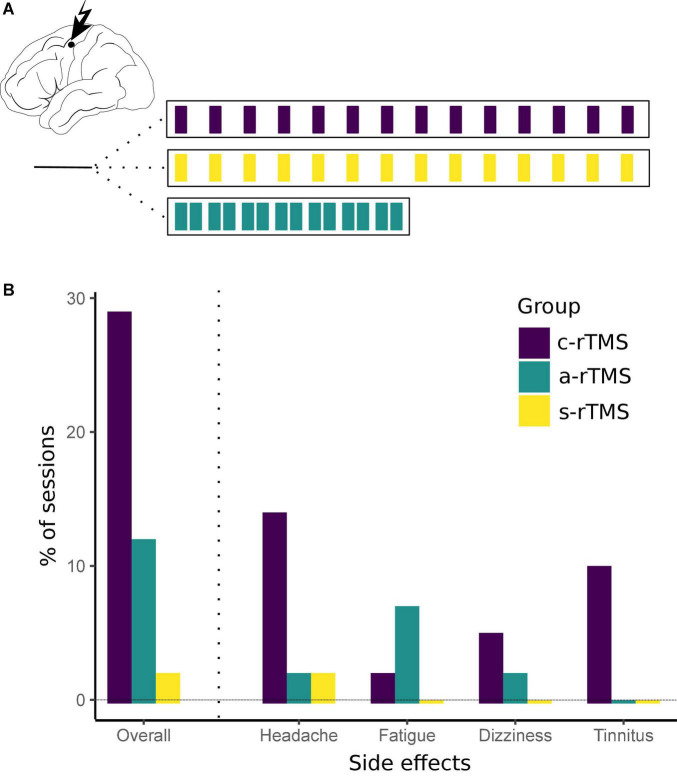
**(A)** Repetitive transcranial magnetic stimulation stimulation conditions. RTMS was applied to the non-dominant primary motor cortex for 30 min per session. Subjects were divided to receive either 14 of days once-daily sham stimulation (s-rTMS), 14 days of once-daily real stimulation (c-rTMS), or 7 days of twice-daily accelerated rTMS (a-rTMS). **(B)** Side effects for each rTMS group. Overall, side effects were of mild intensity and short duration. Most side effects were observed in the c-rTMS group with headaches being the most prevalent condition. Despite intensive screening, one subject in the c-rTMS group reported a chronic tinnitus only after four sessions of rTMS. There was no increase in intensity or incidents of the tinnitus in this subject during the course of the study.

All subjects received 14 sessions of rTMS (1 Hz, 30 min, 1,800 pulses, 110% RMT) to their respective motor hotspot. Subjects were divided evenly into one of three groups: 14 days once daily [classical low-frequency rTMS (c-rTMS)], 7 days twice daily [accelerated rTMS (a-rTMS)], or sham stimulation for 14 days once daily (s-rTMS) (Figure 1A). Daily stimulation sessions in the a-rTMS group were delivered with a 90-min break in between. For sham stimulation, a plastic adapter spacing of 7 cm was placed between the coil and subjects head, thus minimizing the residual electric field reaching the subject’s head to ≤5 μV.

Directly after the stimulation, a motor training of 10 min targeted to the stimulated hand muscles was performed to support reorganization of motor function *via* recruitment of other brain areas. To this purpose, subjects completed exercises such as writing or performing the nine-hole peg test.

### Adverse Event Reporting

After the motor training, subjects were asked to report any side effects of rTMS (“Did you feel any side effects during rTMS?” and “Was anything unpleasant?”). We opted for this open, qualitative question first to not limit subjects in reporting any observation they made during rTMS. Next, they were asked about distinct symptoms (“Did you feel any headache/dizziness/tinnitus?”) to help them identify specific, common side effects of rTMS. Subjects were further asked at the beginning of the next rTMS session if potential side effects had resolved and roughly how long they lasted.

### Data Analysis

We recorded the type of side effect (headache, dizziness, tinnitus, fatigue of stimulated muscles, and others), severity (mild, medium, and severe), and duration (only during rTMS, up to 30 min after rTMS, longer than 30 min). The category ‘‘fatigue of stimulated muscles’’ was added at the analysis stage as it was reported by multiple subjects in the first, open question. Frequency of side effects for each stimulation condition was calculated in RStudio.^1^

## Results

All nine subjects tolerated the stimulation well and completed all 14 rTMS sessions. The RMT (in% of the stimulator output; mean ± SD) in the sham group (36.7 ± 6.7) was higher compared to the active rTMS groups (c-rTMS: 29.3 ± 3.1; a-rTMS group: 28.7 ± 4.6). Subjects reported occurrence of brief headaches in 14% of sessions in the c-rTMS group, 2% in the a-rTMS group, and 0% in the s-rTMS group. Headaches were always reported to be at maximum mild and of short duration (up to 30 min after rTMS). Dizziness during stimulation was reported in 5% of sessions in the c-rTMS group, 2% in the a-rTMS group, and 0% in the s-rTMS. Subjects reported a feeling of fatigue in the stimulated hand muscles in 2% of all sessions in the c-rTMS group, 7% in the a-rTMS group, and 0% in the s-rTMS group. This feeling was noticeable specifically during the consecutive motor training and vanished quickly afterward. None of the subjects in the a-rTMS group reported remaining side effects of the first daily session at the beginning of the second daily session. Of note, despite intensive screening before inclusion, one subject in the c-rTMS group reported a chronic tinnitus pre-dating this study only after four sessions of rTMS. The subject was again informed about the potential of rTMS to trigger or worsen the tinnitus but decided to continue the study. This subject reported a tinnitus on 4 of 14 rTMS sessions, which was comparable to normal days in amount and intensity. In total, 74% of rTMS sessions in the c-rTMS group, 89% in the a-rTMS group, and 98% in the s-rTMS group were free of any side effects. These results are summarized in Figure 1B. When looking at sessions with side effects in the a-rTMS group (Table 1), side effects occurred comparably often during the first daily session (two of five sessions) and the second daily session (three of five sessions).

**TABLE 1:** Number of side effects for each session and group.

	Session													
	1	2	3	4	5	6	7	8	9	10	11	12	13	14
c-rTMS	1	3	1	0	1	1	0	1	1	0	1	1	1	0
a-rTMS	1	0	0	1	0	0	0	1	0	0	0	0	1	1
s-rTMS	0	0	0	0	1	0	0	0	0	0	0	0	0	0

## Discussion

The present pilot study provides preliminary evidence for the safety of accelerated, low-frequency rTMS over the primary motor cortex. Similar to previous studies using shorter single-session durations or lower stimulation intensities (Chae et al., 2004; Flamez et al., 2016), no serious adverse events were observed in this study. The most common adverse events were mild, temporary headaches that disappeared quickly after the end of the stimulation. Side effects did not increase during the second daily stimulation session or with progressing session number, thus suggesting the safety of more intensified stimulation protocols.

As initially argued, accelerated rTMS protocols are needed to make rTMS interventions accessible to a wider range of patients by reducing the treatment duration in days. In the same way, the treatment dose and thereby potentially the efficacy of the intervention can be increased given a certain timeframe. Future studies need to examine this relationship between efficacy of the intervention and treatment duration in days in more detail. Similarly, timings between single rTMS sessions need to be optimized in future studies.

Several limitations of this study should be noted. Side effects were assessed by a short interview rather than standardized questionnaires and severity gradings. We chose this approach so subjects could openly report any observation and, thus, to be open to unexpected side effects that might not be captured with pre-defined questions. However, the reliance mainly on incidence of side effects in this study can be considered as a limitation.

Importantly, the present study aimed to establish preliminary safety of accelerated rTMS in healthy subjects before applying it in potentially more sensitive patient populations. In consequence, our sample only represents a limited group in terms of age and demographic characteristics. Further, we failed to balance our sample regarding subjects’ gender due to local MRI safety regulations excluding women with intrauterine devices from entering the research MRI.

Occurrence of side effects was measured repeatedly within the same subjects. It can be assumed that subjects differed in their pain thresholds and predisposition to report side effects. As we did not assess these cofounding variables prior to randomization, we cannot exclude differences between groups related to these parameters. When looking at the results of this study, it seems that one subject in each experimental group reported a higher incidence of side effects compared to the remaining two subjects in the respective group. Consequently, this highlights the correlation of within-subject reports of side effects, while hinting to a successful randomization of subjects regarding the predisposition to report side effects.

Further, incidence of side effects should theoretically be comparable between both experimental groups as they received the same overall dosage of rTMS. If any differences are observed, then these should be due to a prior daily rTMS session increasing the risk for side effects during the second daily session. In the present study, we found a higher overall incidence of side effects in the classical rTMS compared to the accelerated rTMS group. Further, there was no difference in incidence of side effects between both daily sessions in the a-rTMS group. This might hint to remaining differences in subjects’ predisposition to report side effects, leading to an “over-reporting” of side effects in the c-rTMS group. Conversely, it could also be explained by an “under-reporting” of side effects in the second daily session in the a-rTMS group, for example, due to a more pleasant experience or reduced motivation to report adverse events. In the present study, we cannot differentiate between both potential explanations. Future studies should quantify subjects’ predispositions to report adverse events to control for this confounding variable.

Finally, it should be acknowledged that even mild, temporary side effects should be carefully considered and that subjects should be informed about their potential occurrence. Yet, we argue that, despite the small sample size, this study provides sufficient evidence of safety for other researchers to adapt the protocol in patient samples. In such consecutive studies, larger samples covering different age groups can be acquired and standardized questionnaires can be used to assess safety.

## Data Availability Statement

The raw data supporting the conclusions of this article will be made available by the authors, without undue reservation.

## Ethics Statement

The studies involving human participants were reviewed and approved by Ethikkommission der Charité–Universitätsmedizin Berlin, Campus Charité Mitte, Charitéplatz 1, Berlin. The participants provided their written informed consent to participate in this study.

## Author Contributions

ME: conceptualization, methodology, formal analysis, investigation, data curation, writing–original draft, visualization, and project administration. JK: investigation and writing–review and editing. GR and AC: conceptualization, methodology, and writing–review and editing. TP: conceptualization, methodology, writing–review and editing, and supervision. All authors contributed to the article and approved the submitted version.

## Conflict of Interest

The authors declare that the research was conducted in the absence of any commercial or financial relationships that could be construed as a potential conflict of interest.

## Publisher’s Note

All claims expressed in this article are solely those of the authors and do not necessarily represent those of their affiliated organizations, or those of the publisher, the editors and the reviewers. Any product that may be evaluated in this article, or claim that may be made by its manufacturer, is not guaranteed or endorsed by the publisher.

## Footnotes

1 http://www.rstudio.com/, version 1.3.1073

## Abbreviations

MRI: magnetic resonance imaging
RMT: resting motor threshold
TMS: transcranial magnetic stimulation
rTMS: repetitive TMS
a-rTMS: accelerated twice-daily rTMS
c-rTMS: classical once-daily rTMS
s-rTMS: sham rTMS.
